# The TOFr of 0.75 to 0.85 is the optimal timing for IONM during thyroid surgery: a prospective observational cohort study

**DOI:** 10.1186/s12871-023-02224-8

**Published:** 2023-08-23

**Authors:** Xi Liu, Xue Zhang, Xue Shang, Huihui Wang, Wenting Hou, Zhirong Sun

**Affiliations:** 1 Department of Anesthesiology and Intensive Care Unit, Fudan University Shanghai Cancer Center; Department of Oncology, Shanghai Medical College, Fudan University, Shanghai, China; 2 Department of Anaesthesiology, Shanghai Jiaotong University Affiliated Sixth People’s Hospital, Shanghai, China

## Abstract

**Backgroud:**

Recurrent laryngeal nerve (RLN) injury is one of the serious complications of thyroid tumour surgery, surgical treatment of thyroid cancer requires careful consideration of the RLN and its impact on glottis function. There has been no unified standard for precise neuromuscular block monitoring to guide the monitoring of RLN in thyroid surgery. This study aimed to investigate the correlation between Train-of-four stabilization ratio (TOFr) and neural signal values of intraoperative neurophysiological monitoring (INOM) during thyroid operation, and further to determine the optimal timing for INOM during thyroid operation.

**Methods:**

Patients scheduled for thyroid tumour resection with INOM and RLN monitoring from April 2018 to July 2018 in our center were recruited. Electromyography (EMG) signals and corresponding TOFr were collected. All nerve stimulation data were included in group VR. Vagus nerve stimulation data were included in Subgroup V. RLN stimulation data were included in Subgroup R. The timing of recording was as follows: Vagus nerve EMG amplitude after opening the lateral space between the thyroid and carotid sheath and before the initiation of thyroid dissection, RLN EMG amplitude at first recognition, RLN EMG amplitude after complete thyroid dissection (Repeat three times), and Vagus nerve EMG amplitude after resection of the thyroid (Repeat three times). Correlation analysis of continuous variables was described by a scatter diagram. Pearson correlation analysis or Spearman correlation analysis was used for the two groups of variables.

**Results:**

Finally, 134 vagus nerve signals and 143 RLN signals were analysed after matching with TOFr. The EMG amplitude in the VR group and subgroups after nerve stimulation was positively correlated with TOFr (p < 0.05). In the VR, V and R group, the incidence of EMG ≥ 500 µV in the 0.75 < TOFr ≤ 0.85 interval was significantly higher than the 0 < TOFr ≤ 0.75 interval (P = 0.002, P = 0.013 and P = 0.029), and has no statistical difference compared to 0.85 < TOFr ≤ 0.95 interval (P > 0.05).

**Conclusions:**

The EMG signals of the RLN and vagus nerve stimulation during thyroid surgery were positively correlated with TOFr. TOFr > 0.75 could reflect more than 50% of the effective nerve electrophysiological signals, 0.75 < TOFr ≤ 0.85 interval was the optimal timing for IONM during thyroid surgery.

**Trial registration:**

Chinese Clinical Trial Registry (ChiCTR1800015797) Registered on 20/04/2018. https://www.chictr.org.cn.

## Introduction

Currently, thyroidectomy appears to be the best treatment option for most thyroid tumours [1]. Recurrent laryngeal nerve (RLN) injury is one of the serious complications of thyroid tumour surgery, surgical treatment of thyroid cancer requires careful consideration of the RLN and its impact on glottis function. According to previous studies, the incidence of RLN injury in thyroid surgeries ranges from 0.99 to 13.7% [2–5], and for reoperation, it ranges from 0.6 to 19.4% [6–7]. Regardless of hoarseness caused by unilateral RLN injury or asphyxia induced by bilateral RLN injury, both can significantly impact patients’ lives, leaving a great problem about RLN protection to thyroid surgeons. Previous studies have recommended many techniques and strategies to prevent RLN palsy during thyroid operations [8–9]. IONM is an important technique. Combined with anatomic and functional techniques, IONM has the following advantages: it facilitates the identification and dissection of the RLN, as well as the judgement of functional integrity (10). These features make it a standardized technique that can effectively recognize RLN (2,11) and is accepted by many surgeons, particularly in difficult thyroid operations [12–13].

The application of IONM is restricted by both the location of electromyography (EMG) tube placement and the dose of neuromuscular blocking agent (NMBA) [10, 14]. With the help of the visual laryngoscope, we can easily place the EMG tube in the correct position. However, the residual effect of NMBA not only affects the accuracy of EMG amplitude, but also prolongs the monitoring time. Although the advent of Sugammadex, a specific antagonist, has greatly shortened the recovery time of muscle relaxation of rocuronium [15]. The duration of residual effects of NMBA varies with different doses and classes of muscle relaxation agents [16–17]. Is there a unified standard that would both facilitate the acquisition of accurate EMG amplitudes and save time waiting for monitoring? Many studies have focused on the proper dosage of certain NMBAs to ensure the application of IONM. So far, no research has been undertaken to explore a better anaesthesia strategy for IONM according to precise neuromuscular block monitoring rather than certain NMBA regimens (16–17). Train-of -four stabilization (TOF) is a technique used during general anesthesia to monitor and maintain appropriate levels of muscle relaxation. TOF stimulation involves using a nerve stimulator to deliver a series of four electrical impulses to a peripheral nerve, and observing the results. Train-of -four stabilization ratio (TOFr) is calculated by comparing the amplitude of the fourth muscle contraction in response to the nerve stimulation, to the amplitude of the first muscle contraction. At present,TOFr has been a convenient and routine parameter for neuromuscular block monitoring [18]. This prospective single-center observational study proposes a new strategy that enables us to monitor the RLN timely and effectively in thyroid surgery. The primary goal of this research was to investigate the correlation between TOFr and neural signal values of INOM and determined the optimal timing for intraoperative neurophysiological monitoring (INOM) during thyroid operation.

## Methods

### Ethics statement

This prospective, observational cohort trial was conducted in Fudan University Shanghai Cancer Center. This study protocol was approved by the Ethics Committee of Fudan University Shanghai Cancer Canter, Shanghai, China (Number 1712179-12-1802, Chairperson Prof. Chen Zhen, Approved on 12/02/2018) and registered in the Chinese Clinical Trial Registry (http://www.chictr.org.cn; ChiCTR1800015797, Registered on 20/04/2018). Written informed consent for all forms of personally identifiable data including clinical, and biometric data was obtained from all patients. Any minor or illiterate person participating in the study has obtained the informed consent of a legally authorized representative or guardian. All methods in this study were performed in accordance with the relevant guidelines and regulation.

### Inclusion and exclusion criteria

Both male and female patients scheduled for thyroid tumour resection from April 2018 to July 2018 were recruited and signed the informed consent if they meet the following inclusion criteria: 18–65 years old; American Society of Anaesthesiologists (ASA) physical status I to II; professional head and neck surgeons recommended that patients have indications for IONM; sign the informed consent. All participants in this study were required to sign the informed consent.

Exclusion criteria were as follows: history of epilepsy; history of neurological or neuromuscular disease; history of asthma; predicted difficult airway; preoperative RLN palsies; respiratory insufficiency; without application of the TOF monitor or IONM in the surgery; severe cardiopulmonary diseases; American Society of Anaesthesiologists ≥ 3, pregnant or lactating women; participation in other clinical trials within the past four weeks; and inability to express properly.

### Standard anesthesia procedures

All procedures were performed by the same team of head and neck surgeons, and anaesthesia was administered by two experienced anaesthesiologists and recorded by a trained recorder.

All patients fasted for 6–8 h before surgery. Non-invasive blood pressure, electrocardiography, oxygen saturation, and anaesthesia depth (Narcotrend) were monitored after the patient entered the operating room.

TOFr value was obtained by closed-loop muscle relaxation monitoring system (Guangxi Weilark Technology Co., LTD.), and obtained by stimulating the ulnar nerve of the wrist with an electric current that causes the adductor pollicis response. Before induction, electrodes, muscle tension sensors and temperature sensors should be properly installed as follows: Firstly, 75% alcohol was used to clean the skin. The distal electrode was placed 1 cm from the radial side of the flexor carpi ulnar muscle to the transverse line of the proximal wrist, and the proximal electrode was placed 2.5 cm from the proximal side of the distal electrode. Tape was used to fix the electrode to prevent the electrode from falling off. In addition, the muscle tension sensor is placed in the thumb web space on the same side, and properly fixed with tape to prevent displacement. Finally, place the temperature sensor in the inner pollicis muscle of the palm and secure it with tape to prevent it from falling off. Parameter setting: Stimulation current 50mA, pulse width 200us, the interval of electrical stimulation was 15 s. The TOFr value can be continuously monitored when the start button is pressed, and the muscle relaxation monitoring can be stopped by pressing the pause button.

To reduce measurement bias, the measuring arm will be covered with two layers of cloth to prevent hypothermia of the arm, and the arm will be properly fixed to prevent squeezing or touching.

Before induction of anaesthesia, peripheral venous access was established, and shoulder and head pillows were padded. Anaesthesia induction was initiated with midazolam (Jiangsu Enhua Pharmaceutical Co. Ltd.) 0.05 mg/kg, propofol (Aslikan Pharmaceutical Co. Ltd.) TCI 4 µg/ml, sufentanil (YICHANG humanwell Pharmaceutical Co. Ltd.) 0.2–0.4 µg/kg, remifentanil (YICHANG Humanwell Pharmaceutical Co. Ltd.) TCI 2 ng/ml, rocuronium (Mercadone Co. Ltd.) 0.6 mg/kg. At the same time, mechanically controlled ventilation was started, adjusting respiratory parameters (tidal volume = body weight × [6–8] ml/kg, respiratory frequency 12 times/min, respiratory ratio 1:2). Anaesthesia was maintained with propofol and remifentanil TCI, and the Narcotrend was maintained between 45 and 64.

All patients were intubated with a NIM™ standard enhanced electromyography (EMG) endotracheal tube (Medtronic, Jacksonville, FL) assisted by visual laryngoscopy (7.0 mm internal diameter (ID) for males, 6.0 mm ID for females). The EMG tube electrodes were placed at the level of the vocal cords, and the endotracheal tube was secured. The initial mode was the standby state. The initial respiratory parameters were set as tidal Volume 8 ml/kg (standard body weight), respiratory rate 12 times/min, and respiratory ratio 1:2. The anaesthetic state was maintained with propofol and remifentanil, the Narcotrend was kept between 45 and 64, and the end-tidal carbon dioxide partial pressure (PETCO_2_) was kept between 35 and 45 mmHg.

### Outcome assessment

The NIM 3.0 nerve monitoring system (MedtROnic, Minneapolis, MN, USA) was employed for IONM. EMG signals were obtained with surface electrodes integrated (ETT, NIM Flex EMG tubes, Medtronic) and a handheld bipolar stimulating probe. The current amplitude and impulses have been set at 1 mA and 3 Hz, respectively. The timing of recording was as follows: Vagus nerve EMG amplitude after opening the lateral space between the thyroid and carotid sheath and before the initiation of thyroid dissection(record as V0), RLN EMG amplitude at first recognition(record as R0), RLN EMG amplitude after complete thyroid dissection(Repeat three times, record as Rn), and Vagus nerve EMG amplitude after thyroidectomy(Repeat three times, record as Vn). For those without satisfactory signals, muscle relaxant antagonist (sugammadex 2 mg/kg) was intravenously injected, and signal values were recorded 1 min later. If still not satisfied, the measurement was repeated after a few minutes.

To reduce the deviation caused by measurement timing, The closed-loop muscle relaxation monitoring system and The NIM 3.0 nerve monitoring system were kept on during the operation. Anesthesiologists communicated with the surgeons before each measurement: The electrical stimulation of the RLN or vagus nerve was performed at the same time as the start button of the muscle relaxation monitoring system was pressed. The ulnar nerve stimulation interval is set to 15 s, and if TOFr values and EMG amplitude need to be measured again, the measurement can be repeated after 15 s.When unpaired TOFr and EMG data exist, this set of data will not be included in the analysis. Once TOFr or EMG values were not measured or judged as seriously biased by thyroid surgeons, the corresponding data will not be included in the analysis. All nerve stimulation signal analyses were defined as group VR. Subgroups were defined as follows: Vagus nerve stimulation data were included in Subgroup V. RLN stimulation data were included in Subgroup R; Nerve stimulation in patients with intraoperative sugammadex injection was defined as subgroup S; Nerve stimulation in patients without any muscle relaxant antagonist was defined as subgroup N–S. TOFr was divided into six intervals: TOFr = 0,0 < TOFr ≤ 0.5, 0.5 < TOFr ≤ 0.75, 0.75 < TOFr ≤ 0.85, 0.85 < TOFr ≤ 0.95, and 0.95 < TOFr ≤ 1. When TOFr exceeded 100%, it was classified as a 0.95 < TOFr ≤ 1 interval.

### Statistical analysis

Continuous variables are shown as the means and 95% Confidence interval (95% CI) and were analysed by two-tailed unpaired t tests or one-way ANOVA followed by Bonferroni-corrected pairwise tests. The enumeration data were represented by the numbers of cases (N). Incidences were compared between groups using the chi-square test or Fisher’s exact probability method. Correlation analysis of continuous variables was described by a scatter diagram. Pearson correlation analysis was used for the two groups of continuous variables consistent with a normal distribution, and Spearman correlation analysis was used for the two groups of variables with a nonnormal distribution. All statistical analyses were 2-tailed, and P < 0.05 indicated significant differences. The above statistical methods were also applied to subgroup analysis. Statistical analysis was performed using the SPSS 23.0 software package (IBM Corp, Armonk, NY). The images were produced using the Graphpad prism 8.4.2 software (GraphPad Software,USA).

Several articles ^(19–21)^ have described IONM EMG amplitudes > 500µV as high-quality signals or satisfactory signals. Therefore, we set EMG > 500µV as high-quality EMG amplitude. The pre-experiment results are as follows: Firstly, 0 < TOFr ≤ 0.5, the incidence rate of EMG amplitude ≥ 500 µV for RLN electrical stimulation was 3/15 (20%); TOFr > 0, the incidence of EMG amplitude ≥ 500 µV was 7/15 (46%), α = 0.05,β = 0.2, the number of neural signals required was n = 45. Secondly, TOFr ≤ 0.5, the incidence rate of EMG amplitude ≥ 500 µV by vagus nerve stimulation was 5/15 (30%);TOFr > 0.5, the incidence rate of EMG amplitude ≥ 500 µV by vagus nerve stimulation was 10/15 (0.60%),α = 0.05,β = 0.2, the number of neural signals required is n = 39. Since the same patient can provide electrical stimulation data of the vagus nerve and the RLN, a larger sample size is considered, n = 45. Assuming a dropout rate of 30%, the intraoperative RLN injury rate was 10%, and the dropout rate was 10%. The number of patients needed was calculated to be about 54.

## Results

**Fig. 1:**
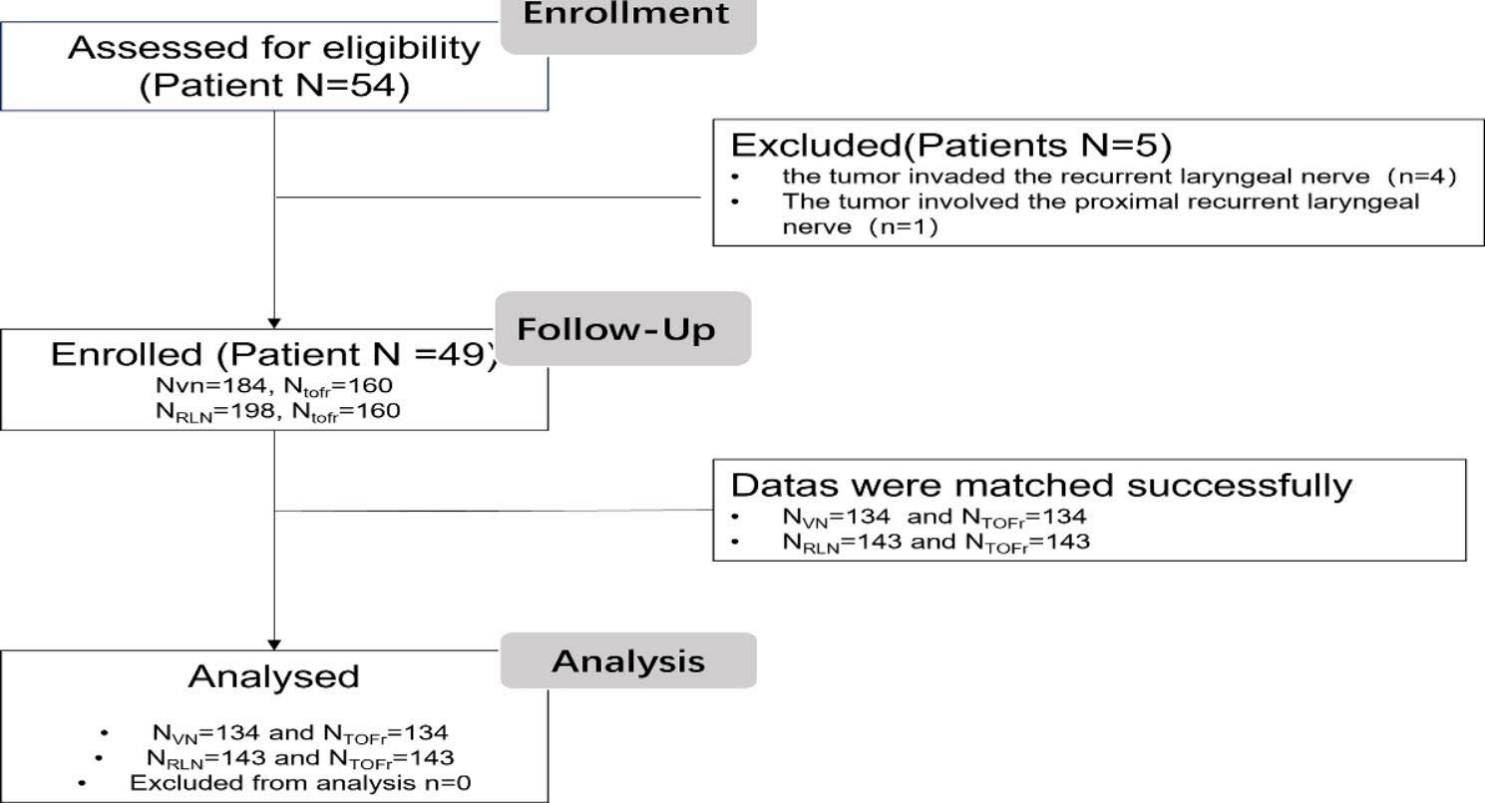
Flow diagram

There were 54 eligible patients scheduled for thyroid tumour resection from April 2018 to July 2018 (Fig. 1]. Five patients were excluded according to the exclusion criteria. Forty-nine patients with 184 Vagus and 198 RLN nerve signals were enrolled in this study. TOFr values that were asynchronous with IONM measurements were excluded. Finally, 134 vagus nerve signals and 143 RLN signals were analysed after matching with TOFr. The demographic data in the study are shown in Table 1.

**Table 1:** Demographic and operation characteristics

Baseline characteristics	Number/mean ± SD
Sex (male/female)(N)	9/40
Age (year)	42.22 ± 10.76
BMI (kg/m^2^)	22.81 ± 3.72
Duration of operation (min)	85.21 ± 44.83
Duration of anesthesia (min)	109.51 ± 45.14
Time interval between induction of anesthesia and initial nerve stimulation(min)	55.67 ± 24.61
Type of thyroid operation	
Total thyroidectomy (left/right/both) (N)	19/17/13
Lymph node excision (left/right/both) (N)	15/18/8
Regional lymph node metastasis(yes/no/ undetected) (N)	18/15/8
Substernal thyroidectomy(N)	1
Papillary thyroid carcinoma/follicular thyroid adenocarcinoma(N)	47/2
TNM staging (American Joint Council on Cancer) < 55year(I/II/III/IVa/IVb)(N) ≥ 55 year(I/II)(N)	44/0 3/2
Number of patients used muscle relaxant antagonists intraoperatively:Sugammadex/ neostigmine(N)	17/32
The hospitalization time(day)	4.72 ± 2.00
TOFr value in subgroup V	0.67 ± 0.36
TOFr value in subgroup R	0.65 ± 0.36
TOFr value in group VR	0.66 ± 0.36

**Fig. 2:**
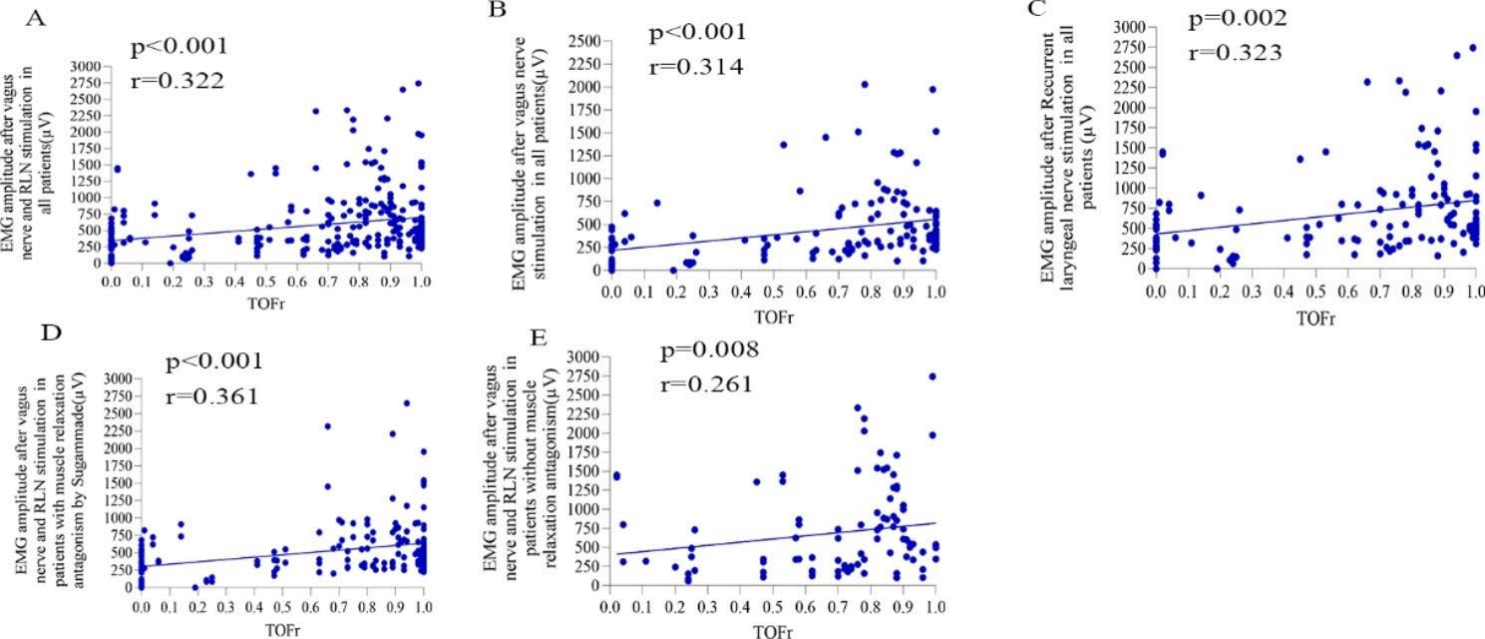
Correlation analysis between nerve stimulation signals and TOFr. (**A**), Correlation analysis between EMG amplitude in the VR and TOFr groups. (**B**), Correlation analysis between EMG amplitude in subgroup V and TOFr. (**C**), Correlation analysis between EMG amplitude in subgroup R and TOFr. (**D**), Correlation analysis between EMG amplitude and TOFr after nerve stimulation in subgroup S. (**E**), Correlation analysis between EMG amplitude and TOFr after nerve stimulation in subgroups N–S

The EMG amplitude in group VR after nerve stimulation was positively correlated with TOFr (p < 0.001,r = 0.322) (Fig. 2].

In subgroup: The EMG amplitude in Group V after vagus nerve stimulation was positively correlated with TOFr (p < 0.001,r = 0.314), the EMG amplitude in Group R after RLN stimulation was positively correlated with TOFr (P = 0.002,r = 0.323). The EMG amplitude after nerve stimulation was positively correlated with TOFr subgroup S (P < 0.001,r = 0.361). The amplitude of EMG after nerve stimulation was positively correlated with TOFr subgroup N–S (P = 0.008,r = 0.261) (Fig. 2].

**Fig. 3:**
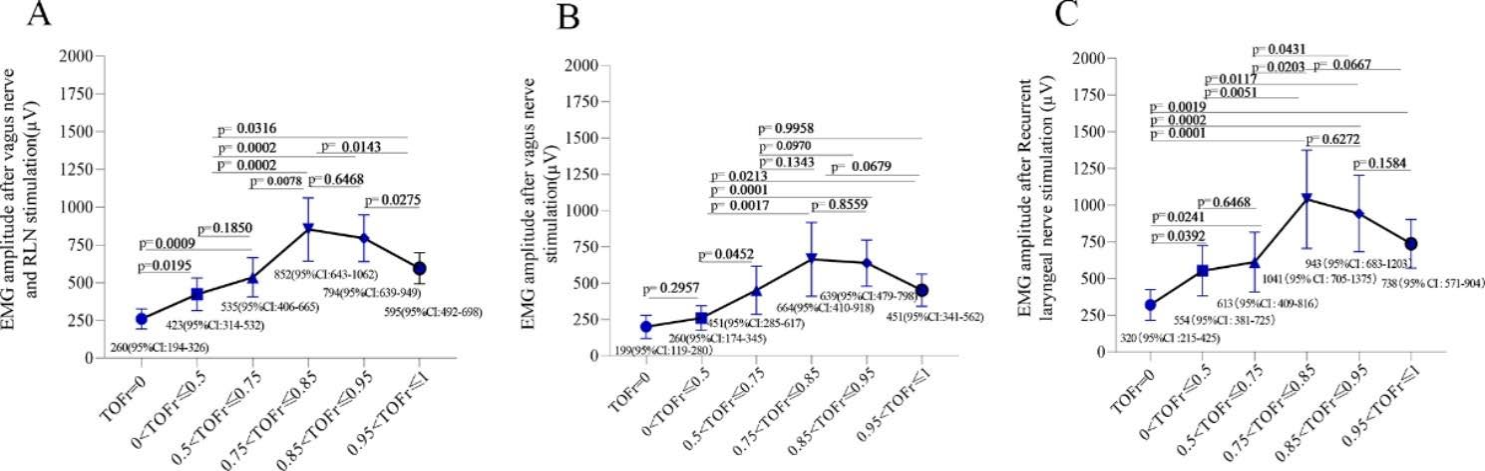
The mean(95%CI) of EMG amplitude of detected nerve stimulation signals in different TOFr intervals. (**A**), The mean(95%CI) of EMG amplitude of detected vagus nerve and RLN stimulation signals in different TOFr intervals. (**B**), The mean(95%CI) of EMG amplitude of detected vagus nerve stimulation signals in different TOFr intervals. (**C**), The mean(95%CI) of EMG amplitude of detected RLN stimulation signals in different TOFr intervals

The mean EMG amplitude of the detected nerve stimulation signals in different TOFr intervals is shown in Fig. 3.

**Fig. 4:**
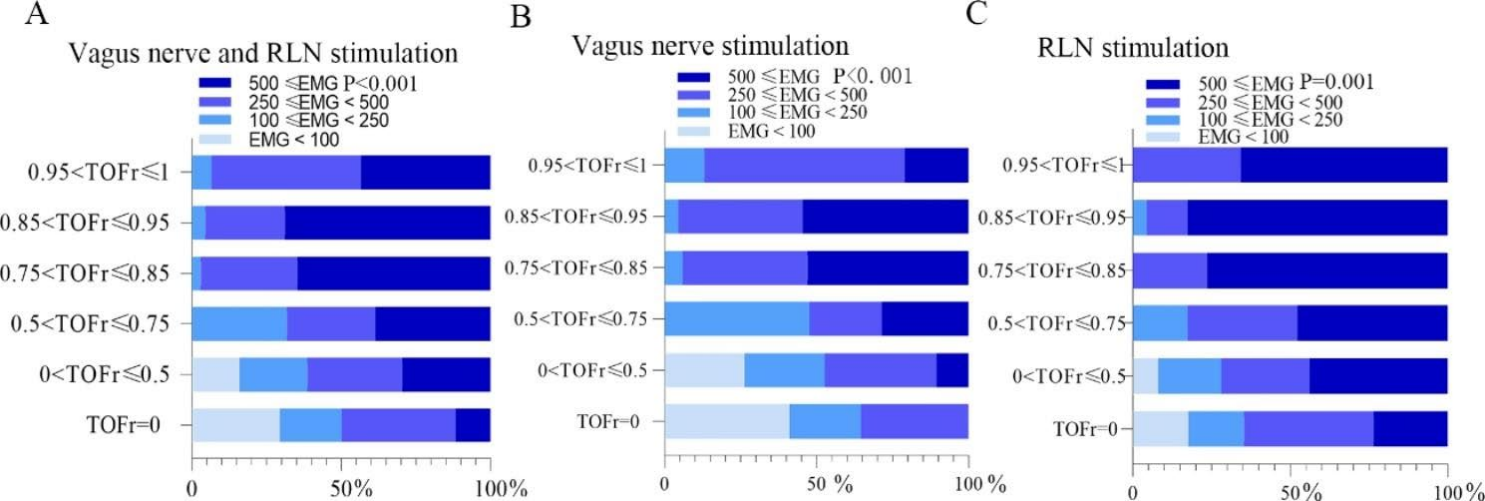
The incidence of EMG amplitudes between different TOFr intervals. (**A**), The incidence of EMG amplitudes between different TOFr intervals for all nerve stimulation signals. (**B)**, The incidence of EMG amplitudes between different TOFr intervals for vague nerve stimulation signals. (**C**), The incidence of EMG amplitudes between different TOFr intervals for RLN stimulation signals. (Coloured circles represent the comparison of rates between the two groups.)

In the VR group (Fig. 3A), the mean amplitude of EMG was close to 500 µV in the 0 < TOFr ≤ 0.5 interval, which is significantly higher than TOFr = 0 (P = 0.0195) and has no significant difference compared with the 0.5 < TOFr ≤ 0.75 interval (P = 0.1850). The incidence of EMG ≥ 500 µV was different among the six TOFr groups by the chi-square test, p < 0.001 (Fig. 4A). The incidence of EMG ≥ 500 µV in the 0.75 < TOFr ≤ 0.85 group was 64.71%, which was significantly higher than that in the 0 < TOFr ≤ 0.75 interval (34.09%) (P = 0.002)(Table 2], and has no statistical difference compared to 0.85 < TOFr ≤ 0.95 interval (P = 0.6468) (Fig. 3A).

**Table 2:** The incidence of EMG amplitude of vagus nerve or RLN stimulation for different TOFr intervals

	EMG < 100 (µV)	100 ≤ EMG < 250 (µV)	250 ≤ EMG < 500 (µV)	250 ≤ EMG (µV)	500 ≤ EMG (µV)
RLN and vagus nerve stimulation,n = 277					
**TOFr = 0,n = 34**	10(29.41%)	7(20.59%)	13(38.24%)	20(58.82%)	4(11.76%)
**0 < TOFr ≤ 0.5,n = 44**	7(15.91%)	10(22.73%)	14(31.82%)	27(61.36%)	13(29.55%)
**0.5 < TOFr ≤ 0.75,n = 44**	0(0)	14(31.82%)	13(29.55%)	30(68.19%)	17(38.64%)
**0.75 < TOFr ≤ 0.85,n = 34**	0(0)	1(2.94%)	11(32.35%)	33(97.06%)	22(64.71%)
**0.85 < TOFr ≤ 0.95,n = 45**	0(0)	2(4.44%)	12(26.67%)	42(93.33%)	31(68.89%)
**0.95 < TOFr ≤ 1,n = 76**	0(0)	5(6.57)	38(50%)	71(93.42%)	33(43.42%)
**vagus nerve stimulation, n = 134**					
**TOFr = 0,n = 17**	7(41.12%)	4(23.53%)	6(35.29%)	6(35.29%)	0(0)
**0 < TOFr ≤ 0.5,n = 19**	5(26.32%)	5(26.32%)	7(36.84%)	9(47.37%)	2(10.53%)
**0.5 < TOFr ≤ 0.75,n = 21**	0(0)	10(47.62%)	5(23.81%)	11(52.38%)	6(28.57%)
**0.75 < TOFr ≤ 0.85,n = 17**	0(0)	1(5.88%)	7(41.18%)	16(94.12%)	9(52.94%)
**0.85 < TOFr ≤ 0.95,n = 22**	0(0)	1(4.55%)	9(40.91%)	21(95.45%)	12(54.55%)
**0.95 < TOFr ≤ 1,n = 38**	0(0)	5(13.16%)	25(65.79%)	33(86.84%)	8(21.05%)
**RLN stimulation,n = 143**					
**TOFr = 0,n = 17**	3(17.64%)	3(17.64%)	7(41.18%)	11(64.71%)	4(23.53%)
**0 < TOFr ≤ 0.5,n = 25**	2(8%)	5(20%)	7(28%)	18(72.00%)	11(44.00%)
**0.5 < TOFr ≤ 0.75,n = 23**	0(0)	4(17.39%)	8(34.78%)	19(82.60%)	11(47.83%)
**0.75 < TOFr ≤ 0.85,n = 17**	0(0)	0(0)	4(23.53%)	17(100.00%)	13(76.47%)
**0.85 < TOFr ≤ 0.95,n = 23**	0(0)	1(4.35%)	3(13.04%)	21(91.30%)	19(82.61%)
**0.95 < TOFr ≤ 1,n = 38**	0(0)	0(0)	13(34.21%)	38(100.00%)	25(65.79%)

In subgroup V (Fig. 3B), the mean EMG amplitude was close to 500 µV in the 0.5 < TOFr ≤ 0.75 interval, which was significantly higher than that in the 0 < TOFr ≤ 0.5 interval (P = 0.0452). The incidence of EMG ≥ 500 µV was different among the six TOFr groups by the chi-square test, p < 0.001 (Fig. 4B). The incidence of EMG ≥ 500 µV in the 0.75 < TOFr ≤ 0.85 group was 52.94%, which was significantly higher than that in the 0 < TOFr ≤ 0.75 interval (9.09%) (P = 0.013) (Table 2], and has no statistical difference compared to 0.85 < TOFr ≤ 0.95 interval (P = 0.8559) (Fig. 3B).

In subgroup R (Fig. 3C), the incidence of EMG amplitudes exceeding 500 µV in the interval 0 < TOFr ≤ 0.5 was significantly greater than that of TOFr = 0(p = 0.0392). the incidence of EMG ≥ 500 µV was different among the six TOFr groups by the chi-square test, p = 0.001 (Fig. 4C). The incidence of EMG ≥ 500 µV in the 0.75 < TOFr ≤ 0.85 group was 76.47%, which was significantly higher than that in the 0 < TOFr ≤ 0.75 interval (45.83%) (P = 0.029)(Table 2], and has no statistical difference compared to 0.85 < TOFr ≤ 0.95 interval (P = 0.6272) (Fig. 3C).

## Discussions

RLN paralysis continues to be a major source of morbidity after thyroid surgery [8, 22−23] and can negatively affect the patient’s quality of life. All causes have brought about RLN injury becoming the primary reason for medical litigations in many countries in recent years [24]. Therefore, avoiding injury to the RLN is one of the key factors for the success of thyroid tumour surgery. IONM has been widely accepted as a gold-standard adjunct for visual identification of the RLN during thyroid surgery [5, 25]. Multiple studies have shown that IONM use during thyroidectomy is associated with a reduced risk of RLN injury [26–28]. Before thyroid dissection, to prevent RLN injury, it is necessary to first identify RLN. Despite controversial studies ^(8)^, IONM during surgery can improve nerve identification rates [26–28], the identification rate of RLN with IONM is approximately 99.3%, in contrast to 90% without IONM (29–30). Although IONM has been used more widely in clinical practice, many factors can cause misleading information [31–32], which may place great psychological pressure on surgeons, delay the surgical process, and even cause surgeons to make incorrect decisions. Muscle relaxants are one of the affecting factors [14, 18]. During the past decade, studies have focused on proper anaesthesia strategies for the application of IONM. Five NMBA regimens for IONM could be concluded: (a) relaxant-free regimen, (b) succinylcholine, (c) titration of nondepolarizing NMBA, and(d) rocuronium combined with sugammadex [14]. Therefore, many studies have experimented with muscle relaxants to obtain a proper dose to reduce the effect on IONM [14, 17−18]. Although the neuromuscular block effect of rocuronium could be rapidly reversed by sugammadex, the recovery time of muscle relaxation was different with different doses of sugammadex. In addition, some procedures use other neuromuscular blockers with or without the use of muscle relaxation antagonists. How to determine the best timing of nerve stimulation to reduce the waiting time and obtain effective EMG signals for nerve stimulation? Our aim was to explore the best timing for developing a general neuromuscular block monitoring strategy to guide the use of IOMN.

It is well known that NMBAs can affect the movement of laryngeal muscles. However, Marusch [33] demonstrated that the relaxation of the adductor pollicis muscle and the vocalis muscle were significantly different. Laryngeal muscles need less recovery time than adductor pollicis muscles from the same relaxation degree. However, TOF was calculated according to the relaxation degree of the adductor pollicis muscle. Is there a correlation between the TOFr and the degree of relaxation of vocal muscles? What is the correlation between TOFr and EMG amplitude? In most studies of thyroid surgery, the optimal timing of nerve stimulation has not been analysed, although intraoperative muscle relaxation monitoring has been used primarily to guide the use of neuromuscular blockers or muscle relaxation antagonists. To explore whether nerve stimulation signals vary with TOFr values, we compared EMG amplitudes between different TOFr values. Analysis showed that EMG amplitude after nerve stimulation (vagus and RLN) was positively correlated with TOFr (P = 0.002,r = 0.323). After subgroup analysis, the amplitude of EMG was positively correlated with TOFr in the four subgroups, including vagus nerve stimulation(p < 0.001,r = 0.314), RLN stimulation(P = 0.002,r = 0.323), intraoperative nerve stimulation with sugammadex(P < 0.001,r = 0.361) and intraoperative nerve stimulation without muscle relaxant antagonist (P = 0.008,r = 0.261). Therefore, the TOF ratio can reflect the relaxation degree of vocal muscles with complete nerve conduction, and the degree of change in the nerve signal is positively correlated with the TOFr value.

As shown in Fig. 3, the mean EMG amplitude of electrical stimulation of vagus nerve was lower than that of RLN in each TOFr interval. To achieve the same mean EMG amplitude (≥ 500 µV), TOFr > 0.5 is required for RLN, but TOFr > 0.75 is required for vagus nerve. The possible reason is that the vagus nerve has a larger inner diameter than the recurrent laryngeal nerve and requires stronger electrical stimulation to achieve the same EMG amplitude.

When TOFr was ≤ 0.85, the mean RLN or vagus nerve EMG amplitude increased with increasing TOFr. Once TOFr > 0.85, the mean EMG amplitude did not increase with the increase of TOFr. There was a significant difference in the degree of relaxation of the adductor pollicis muscle and the vocalis muscle. The laryngeal muscles exhibited a shorter response time than the adductor pollicis and recovered more quickly [33–34.). When the TOFr of adductor pollicis reached 0.85, the TOFr of laryngeal muscles was larger and even recovered completely. For patients using micuronium ^(34)^, the time from injection to recovery of the twitch response to 75% and 90% was 25.3 Vs 13.8 min and 27.4 Vs 15.5 min, respectively.

When TOFr is greater than 0.75, it could reflect more than 76.47% of the effective RLN electrophysiological signals and reflect more than 52.94% of the effective Vagus nerve electrophysiological signals. When TOFr is less than 0.75, it could only reflect less than 38.64% of the effective Vagus nerve electrophysiological signals. Considering the mean EMG amplitudes and the incidence of EMG amplitudes > 500 µV in different TOFr intervals, TOFr less than 0.75 can still be appropriate for IONM, but 0.75 < TOFr ≤ 0.85 was the optimal timing for IONM during thyroid surgery. For 0.95 < TOFr ≤ 1 interval, both the mean EMG amplitude and the incidence of EMG > 500 µV were smaller than 0.75 < TOFr ≤ 0.85 interval. The reasons may be confounding factors. First, data were missed due to machine connection or probe connection problems. Second, there may be potential minor nerve injury during thyroidectomy.

There is more than one method to assess laryngeal muscle function [11]. Adduction of the vocal cords was detected by the electromyography–endotracheal tube and abduction by finger palpation of muscle contraction in the posterior cricoarytenoid ^(11)^. Therefore, by utilizing both techniques, both the adductor and abductor function of the larynx can be assessed. Should optimal TOFr for IONM be evaluated using more objective methods? Chan WF ^(35)^ validated the ability of IONM to predict the postoperative functional outcome of RLN during thyroid surgery: the sensitivity, specificity, and positive and negative predictive values were 53%, 94%, 35%, and 97%, respectively. With the development of IONM technology, sensitivity, specificity, positive and negative predictive value, and accuracy, respectively, were 100%, 94%, 100%, 97%, and 98% for diagnosis of RLN ^(36)^. Tomoda ^(37)^ examined the sensitivity and specificity of the laryngeal palpation test. For postoperative vocal cord palsy, the sensitivity, specificity and positive and negative predictive values of the laryngeal palpation test were 69.3%,99.7%, 92.1% and 98.5% respectively. For permanent vocal cord palsy, the sensitivity, specificity and positive and negative predictive values were 85.7%, 97.3%, 23.7% and 99.8% respectively. Laryngeal palpation is useful as an adjunct to formal EMG monitoring during thyroid and parathyroid surgery. Thus, Laryngeal palpation can be used as an auxiliary method to evaluate optimal TOFr.

There were several limitations to this study: Firstly, previous studies recommended a stimulator probe current intensity of 1–2 mA [38]. Many other studies choose 2 mA to map the RLN and 1 mA or less to verify the path of the nerve or to distinguish the nerve from other tissues [39–40]. However, in our study, we only used 1 mA, which is the standard setting of the IONM machine and is normally accepted for daily work. Since the inner diameter of the vagus nerve is larger than RLN, the use of 1mA electrical stimulation of the vagus nerve may result in false positives, which is the limitation of this study. Secondly, this study excluded patients with a history of neurological or neuromuscular diseases, respiratory insufficiency, and severe cardiopulmonary diseases, etc. Whether the conclusions of this study are applicable to this population needs further exploration. Thirdly, The EMG and elicited TOFr values may be biased in timing. Although the anesthesiologist and surgeons have communicated enough to ensure that the two machines deliver electrical stimulation at the same time, there may still be a time lag of several seconds. To ensure that the muscle has fully recovered from the previous stimulation and that the results are accurate, we set the interval of electrical stimulation of the ulnar nerve to 15s. Although the muscle relaxation monitoring equipment was fixed before surgery, the EMG and TOFr values could not be matched due to extrusion, electrode shedding and other reasons, and repeated measurements were required. Finally, this study did not assess the effect of inhaled anesthetics on IONM. Inhalation anesthesia was not used for anesthesia maintenance in this study. Studies ^(41–42)^ have shown that Inhaled anesthetics can produce muscle relaxation and prolong the recovery time of TOF, their synergies with muscle relaxants increase correspondingly with increasing concentration and time. Does inhalation anesthesia affect the relationship between IONM signal values and TOFr during thyroid surgery? A randomized controlled trial^(43)^ concluded that combined intravenous and inhaled anesthesia based on sevoflurane-remifentanil prolonged the time until detection of a positive EMG signal during IONM as compared with TIVA with propofol-remifentanil in patients undergoing thyroidectomy. IONM has been shown to reduce the incidence of RLN injury in patients undergoing a second thyroid operation, even when anesthesia is maintained with intravenous combined inhalation anesthesia (sevoflurane) ^(44)^. In conclusion, inhaled anesthetics prolong the time for TOFr to recover to a certain value, but there is no evidence that inhaled anesthetics influence IONM signal value and TOFr.

## Conclusions

The electromyogram signals of the RLN and vagus nerve stimulation during thyroid surgery were positively correlated with TOFr. When TOFr is greater than 0.75, it could reflect more than 50% of the effective nerve electrophysiological signals, 0.75 < TOFr ≤ 0.85 was the optimal timing for IONM during thyroid surgery.

## Data Availability

The data sets used and/or analyzed during the present study are available from the corresponding author on reasonable request. The emails of corresponding author: sunrongsun@shca.org.cn; 1,521,780,363@qq.com; whh_mz@aliyun.com.

## Abbreviations

RLN: Recurrent laryngeal nerve
IONM: Intraoperative neurophysiological monitoring
TOFr: Train-of -four stabilization ratio
ASA: American Society of Anaesthesiologists
EMG: Electromyography
